# Genes responsible for proliferation, differentiation, and junction adhesion are significantly up-regulated in human ovarian granulosa cells during a long-term primary in vitro culture

**DOI:** 10.1007/s00418-018-1750-1

**Published:** 2018-10-31

**Authors:** Wiesława Kranc, Maciej Brązert, Joanna Budna, Piotr Celichowski, Artur Bryja, Mariusz J. Nawrocki, Katarzyna Ożegowska, Maurycy Jankowski, Błażej Chermuła, Marta Dyszkiewicz-Konwińska, Michal Jeseta, Leszek Pawelczyk, Andrzej Bręborowicz, Dominik Rachoń, Małgorzata Bruska, Michał Nowicki, Maciej Zabel, Bartosz Kempisty

**Affiliations:** 1 Department of Anatomy, Poznan University of Medical Sciences, 6 Święcickiego St, 60-781 Poznań, Poland; 2 Division of Infertility and Reproductive Endocrinology, Department of Gynecology, Obstetrics and Gynecological Oncology, Poznan University of Medical Sciences, 33 Polna St, 60-535 Poznań, Poland; 3 Department of Histology and Embryology, Poznan University of Medical Sciences, 6 Święcickiego St, 60-781 Poznań, Poland; 4 Department of Biomaterials and Experimental Dentistry, Poznan University of Medical Sciences, 70 Bukowska St, 60-812 Poznań, Poland; 5 Department of Obstetrics and Gynecology, University Hospital and Masaryk University, Jihlavská 20, 625 00 Brno, Czech Republic; 6 Department of Pathophysiology, Poznań University of Medical Sciences, 8 Rokietnicka St, 60-806 Poznan, Poland; 7 Department of Clinical and Experimental Endocrinology, Medical University of Gdańsk, 7 Dębinki St, 80-211 Gdańsk, Poland; 8 Division of Histology and Embryology, Department of Human Morphology and Embryology, Wroclaw Medical University, Wroclaw, Poland; 9 Division of Anatomy and Histology, University of Zielona Góra, Zielona Góra, Poland

**Keywords:** Granulosa cells, Proliferation, Differentiation, Stem cells, Microarrays

## Abstract

The human ovarian granulosa cells (GCs) surround the oocyte and form the proper architecture of the ovarian follicle. The ability of GCs to proliferate and differentiate in the conditions of in vitro culture has been proven. However, there is still a large field for extensive investigation of molecular basics, as well as marker genes, responsible for these processes. This study aimed to find the new marker genes, encoding proteins that regulate human GCs in vitro capability for proliferation and differentiation during long-term primary culture. The human follicular GCs were collected from hyper-stimulated ovarian follicles during IVF procedures and transferred to a long-term in vitro culture. The culture lasted for 30 days, with RNA samples isolated at days 1, 7, 15, 30. Transcriptomic analysis was then performed with the use of Affymetrix microarray. Obtained results were then subjected to bioinformatical evaluation and sorting. After subjecting the datasets to KEGG analysis, three differentially expressed ontology groups “cell differentiation” (GO:0030154), “cell proliferation” (GO:0008283) and “cell–cell junction organization” (GO:0045216) were chosen for further investigation. All three of those ontology groups are involved in human GCs’ in vitro lifespan, proliferation potential, and survival capability. Changes in expression of genes of interest belonging to the chosen GOs were validated with the use of RT-qPCR. In this manuscript, we suggest that *VCL, PARVA, FZD2, NCS1*, and *COL5A1* may be recognized as new markers of GC in vitro differentiation, while *KAT2B* may be a new marker of their proliferation. Additionally, *SKI, GLI2, FERMT2*, and *CDH2* could also be involved in *GC* in vitro proliferation and differentiation processes. We demonstrated that, in long-term in vitro culture, GCs exhibit markers that suggest their ability to differentiate into different cells types. Therefore, the higher expression profile of these genes may also be associated with the induction of cellular differentiation processes that take place beyond the long-term primary in vitro culture.

**Electronic supplementary material:**

The online version of this article (10.1007/s00418-018-1750-1) contains supplementary material, which is available to authorized users.

## Introduction

Folliculogenesis and oogenesis start in the early embryo and continue until the very end of the reproductive period. During fetal life, the primordial follicles are formed. A primordial follicle contains the oocyte (arrested in the prophase of the first meiotic division), surrounded one layer of flat follicular cells and basal lamina. Successive stages of oogenesis and folliculogenesis occur after birth. During folliculogenesis, secondary and tertiary follicles are formed. From puberty to menopause, every month, one follicle usually matures. Before ovulation, four types of granulosa cells (GCs) of common origin: theca interna, mural granulosa cells, cumulus oophorous, and corona radiata, are located inside the mature (preovulatory) follicle (Chachuła et al. 2016; Rybska et al. 2018). While their structural function in follicular architecture is prominent, recent studies also suggested that ovarian GCs may display a more important role in the follicular development of the oocytes (Gao et al. 2014; Kranc et al. 2016, 2017a). Under physiological conditions, GCs have an inhibitory effect on the meiosis process occurring in the oocyte, affecting oocytes through the follicular fluid (Tsafriri and Channing 1975; Kranc et al. 2018). It was shown that early denuded oocytes do not finish their nuclear and/or cytoplasmic maturation, which needs to be completed before fertilization (Tanghe et al. 2002; Kranc et al. 2017c). Experiments on porcine GCs indicated that these cells manifest a significant expression of gap junction connection (GJC) genes and proteins (Antosik et al. 2010; Kempisty et al. 2014b). That data implied that not only CCs but also GCs may mediate the somatic cell–oocyte “dialogue” and permanent transport of small metabolites, necessary for the support of proper growth and development of the gamete (Dumesic et al. 2015; Russell et al. 2016; Budna et al. 2017). Hence, it is suggested that human GCs are more than remnant material, which is often disposed of during in vitro procedures in animals and humans, and could instead serve in potential research and clinical applications. Therefore, we have used microarrays to search for the new marker genes involved in junction connections and cellular adherence in human ovarian GCs during their long-term in vitro culture. These experiments may bring a new light into the function of human GCs as the cells involved in important somatic cell–oocyte connections, as well as mediators in metabolic pathways crucial for the growth of both oocytes and the somatic cells themselves.

Experiments performed on porcine GCs indicated that these cells are characterized by an increased ability for proliferation during in vitro culture. It was also well-recognized that GCs display significant expression of luteinizing hormone (LH), follicle-stimulating hormone (FSH) and P450 aromatase, which belong to markers of GC luteal differentiation, in the first 48/72 h of short-term in vitro culture (Kempisty et al. 2015). The in vitro proliferation of porcine GCs was substantially accompanied by changes in the transcriptomic profile (unpublished data). Therefore, it is suggested that GCs undergo significant biochemical modifications in long-term primary culture, which may also be a sign of cellular differentiation. Kossowska-Tomaszczuk et al. have recently shown that human GCs may be induced to differentiate into chondroblasts, osteoblasts, and neuroblasts, if placed in a long-term in vitro culture based on specifically supplemented differentiating medium (Kossowska-Tomaszczuk et al. 2009). These experiments indicated a huge stem-like specificity and plasticity of human GCs in primary culture. These results label GCs as potential candidates for future use as allografts in regenerative and reconstructive medicine (Jankowski et al. 2018; Kranc et al. 2018). In this study, we aim to recognize the expression patterns of genes involved in their in vitro proliferation and differentiation. These genes could potentially be used as markers of in vitro developmental capability of human GCs.

## Materials and methods

### Patients and collection of granulosa cells

The GCs were derived from patients undergoing in vitro fertilization (IVF), who had given their informed, written consent to participate in the research and be included in this protocol. The study group consisted of 8 patients, aged 18–40 years, with diagnosed infertility, referred to the Division of Infertility and Reproductive Endocrinology, Poznan University of Medical Sciences, Poland. The procedure was based on controlled ovarian hyperstimulation protocol, adjusted to the patient’s initial infertility workup and ovarian response. Stimulation was performed using recombinant FSH (Gonal-F, Merck Serono) and highly purified hMG-HP (Menopur, Ferring). Cetrorelix acetate injections (Cetrotide, Merck Serono) were administered in an adequate dose, to suppress pituitary function. Ovulation triggering was based on a subcutaneous injection of 6500 U of hCG (Ovitrelle, Merck-Serono). Follicular fluid, containing GCs, was collected during transvaginal, ultrasound-guided oocyte pick-up, 36 h after human chorionic gonadotropin administration. The content of follicles that were over 16 mm in diameter was passed immediately to an embryologist, who isolated the oocyte and pooled the fluids containing GCs together from each ovary. The fresh follicular fluid was centrifuged for 10 min at 200 g, to separate and collect GCs. Patients with polycystic ovary syndrome (PCOS), endometriosis, and diminished ovarian reserve (serum anti-müllerian hormone less than 0.7 ng/mL, and/or day 2–3 FSH serum level higher than 15 mU/mL, and/or antral follicle count less than 9) were excluded from the study. The research has been approved, with resolution 558/17, by Poznan University of Medical Sciences Bioethical Committee.

### Primary cell culture

The collected cells were washed twice by centrifugation (200×*g*, 10 min at RT), using culture medium. Medium consisted of Dulbecco’s Modified Eagle’s Medium (DMEM, Sigma-Aldrich, USA), 2% fetal bovine serum FBS (FBS; Sigma-Aldrich Co., St. Louis, MO, USA), 200 mM l-glutamine, 10 mg/ml gentamicin 10,000 units/ml penicillin, and 10,000 µg/ml streptomycin (all Invitrogen, Carlsbad, CA, USA). Cells were cultivated at 37 °C under aerobic conditions (5% CO_2_). Once adherent cells were more than 90% confluent, they were detached by applying 0.05% trypsin–EDTA solution (Invitrogen, USA) for 1–2 min and counted using a “Neubauer improved” chamber counting (ISO LAB Laborgerate GmbH, DIN EN ISO CERTIFIED 9001). GCs were then cultivated for 30 days. The medium was changed twice a week. Finally, total RNA was isolated from the cells after 1, 7, 15 and 30 days of IVC.

### Immunofluorescence analysis of LHR and FSHR expression

To confirm their identity, GCs were transferred to 35 mm bottom glass dishes (MatTek Corporation, Ashland, USA) according to the normal procedure described above. After 1, 7 and 30 days of culture, the cells were fixed in 4% paraformaldehyde (PFA) solution for 10 min at room temperature (RT). To block non-specific binding, samples were incubated in 3% BSA in PBS for 30 min at RT. Then, they were incubated for 1 h at RT with the rabbit monoclonal anti-luteinizing hormone receptor antibody (anti-LHR, ab103874; Abcam, Cambridge, UK) or goat polyclonal anti-follicle-stimulating hormone receptor antibody (anti-FSHR, sc-26341; Abcam, Cambridge, UK), diluted 1:500 in PBS. Afterwards, the samples were rinsed with PBS and incubated for another 1 h in RT with fluorescein isothiocyanate (FITC)-conjugated donkey anti-goat IgG (MFP488, MoBiTech GmbH, Göttingen, Germany) and/or goat anti-rabbit IgG (ab6717, Abcam, Cambridge, UK), respectively, diluted 1:500 in PBS. Finally, the samples were stained with 0.1 µg/ml 4,6-diamino-2-phenylindole (DAPI; Santa Cruz Biotechnology, Santa Cruz, CA, USA) and examined under confocal Fluoview 10i microscope (Olympus, Tokyo, Japan). FITC was excited with an argon laser at 488 nm, with emissions imaged through a 505–530 nm filter. All the resulting images were analyzed using Imagis 7.2 Software (BitPlane, Zurich, Switzerland).

### Total RNA isolation

The modified Chomczyński–Sacchi method was used to isolate the total RNA (Chomczynski and Sacchi 1987). The GCs were suspended in 1 ml of guanidine thiocyanate and phenol mixture in monophase solution (TRI Reagent^®^, Sigma-Aldrich, USA, St. Luis). After further addition of chloroform, the sample was centrifuged until three visible phases were obtained. RNA was located in the upper, aqueous phase. The resulting RNA was intact, with little or no contaminating DNA and protein. The last step was the stripping of RNA with 2-propanol (Sigma-Aldrich, USA, St. Luis, catalogue number I9516), added in an amount appropriate for 1 mL of TRI reagent used, followed by washing with 75% ethanol. RNA prepared in that way was used for further analysis.

### Microarray expression analysis and statistics

To investigate the transcriptomic changes, whole gene expression analysis was performed with the use of Affymetrix^®^ Human Genome U219 Array (Affymetrix, Santa Clara, CA, USA). Total RNA (100 ng) from each pooled sample was subjected to two rounds of sense cDNA amplification (Ambion^®^ WT Expression Kit; Ambion Inc., Foster City, CA, USA). The obtained cDNA was used for biotin labelling and fragmentation by Affymetrix GeneChip^®^ WT Terminal Labeling and Hybridization. Biotin-labelled fragments of cDNA (5.5 µg) were hybridized to the Affymetrix^®^ Human Genome U219 Array (48 °C/20 h). Microarrays were then washed and stained, according to the technical protocol, using the Affymetrix GeneAtlas Fluidics Station. The array strips were scanned employing the Imaging Station of the GeneAtlas System. Preliminary analysis of the scanned chips was performed using Affymetrix GeneAtlasTM Operating Software (all Affymetrix, Santa Clara, CA, USA). The quality of gene expression data was confirmed according to the control criteria provided by the software. The obtained CEL files were imported into downstream data analysis software. All the presented analyses and graphs were plotted using Bioconductor and R programming languages. Each CEL file was merged with a description file. Subsequently, the Robust Multiarray Averaging (RMA) algorithm was used to correct background, as well as normalize and summarize the results. Statistical significance of the analyzed genes was determined using moderated *t* statistics from the empirical Bayes method. The obtained *p* value was corrected for multiple comparisons using Benjamini and Hochberg’s false discovery rate. The selection of significantly altered genes was based on a *p* value beneath 0.05 and expression change higher than twofold. The list of differentially expressed genes (separated into up- and down-regulated groups) was uploaded to the DAVID (Database for Annotation, Visualization, and Integrated Discovery) software (Huang et al. 2007). Selected genes were input into the STRING (Search Tool for the Retrieval of Interacting Genes/Proteins) software, to analyze their predicted interactions. STRING is a huge database containing information on protein/gene interactions, including experimental data, computational prediction methods, and public text collections (von Mering et al. 2004). The predictions were based on text mining, co-expression, and experimentally observed interactions.

The representation of differently expressed genes in “adherens junction” and “tight junction” KEGG pathways was marked using Pathview Bioconductor package (Luo and Brouwer 2013).

To further investigate the chosen genes belonging to “cell differentiation” (GO:0030154), “cell proliferation” (GO:0008283) and “cell–cell junction organization” (GO:0045216) GO terms, their mutual relations were investigated using the GOplot package (Walter et al. 2015). The GOplot package also allowed for calculation of the *Z* score (the number of up-regulated genes minus the number of down-regulated genes divided by the square root of the count). The *Z* score analysis allowed for the comparison of the enrichment of selected GO BP terms.

### Real-time q-PCR analysis

The RT-qPCR was used to confirm the results obtained through expression microarrays. Three genes showing the highest, lowest, and intermediate level of expression were selected from each heatmap. Changes in the level of expression of those genes were then examined. Three biological samples of each gene were used for the analysis. Each biological test was performed in three technical replicates. Reverse transcription was based on the protocols and reagents of SABiosciences (RT^2^ First Stand Kit -330401), using a Veritimer 96 well Thermal Cycler. 1 µg of each gene’s RNA transcript was used for reverse transcription. Real-time PCR was performed using the 7900HT Fast Real-Time PCR System (Applied Biosystems), RT^2^ SYBR^®^ Green ROX™ qPCR Master Mix (Qiagen Sciences, Maryland, USA), and sequence-specific primers (Table 1). *Glyceraldehyde-3-phosphate dehydrogenase* (*GADPH*), *β-actin* (*ACTB*), and *hypoxanthine phosphoribosyltransferase 1* (*HRPT1*) were used as reference genes. Gene expression was analyzed using the relative quantification (RQ) method. The q-PCR starters were designed using Primer3Plus software (http://primer3plus.com/cgi-bin/dev/primer3plus.cgi). The sequences of the respective genes were taken from the Ensembl database (http://www.ensembl.org/index.html), from which only the sequence of exons was exported, as the target sequence of the designed starter was spread across the border of two adjacent exons. This approach was used as a precaution against the possibility of a non-specific DNA template-based product (DNAse contained in the reverse transcription kit that was used as the other precaution). The primer attachment temperature was the geometric mean of all used primers.

**Table 1:** Primers. Oligonucleotide sequences of primers used for RT-qPCR analysis

Gene	Gene accession numer	Primer sequence (5′–3′)	Product size (bp)
CSRP3	NM_003476	ACAGGCAGACTTGACCTTGAC TCACAGGCTCCACATTTTGC	77
FZD2	NM_001466	TTCCACCTTCTTCACTGTCACC AGCAGCCCGACAGAAAAATG	89
GADD45B	NM_015675	TGATGAATGTGGACCCAGACAG TGAGCGTGAAGTGGATTTGC	96
SPAG16	NM_024532	GTTGGGCAGATTTCTGGACTTC TGGCTTCACGAAGACCTTTC	145
OSR1	NM_145260	TTCAGCTAAAGCCCCAGAGAC TGGCTTCTCAATCCGGATCTTG	70
ITGA6	NM_001079818	TTTATCGGTCTCGGGAGTTGC ATAGCTTGCTCGCCAACAAC	72
EDN2	NM_001956	TGTTCCAGACTGGCAAGACAG TGACTGTGGAAATGTCCCTCAG	72
TESC	NM_017899	TCAGCCTACCATTCGCAAGG TTGTCGAAGAAGGCACGAAC	93
TGFBR1	NM_004612	AGAGCTGTGAAGCCTTGAGAG TTCCTGTTGACTGAGTTGCG	122
CDC6	NM_001254	TCAATTCTGTGCCCGCAAAG TAGCTCTCCTGCAAACATCCAG	74
LIMS1	NM_001193482	GTGGCATGTGGAGCATTTTG AACACGATTGCAGTGGAAGC	142
PKP2	NM_004572	ATGCTAAAGGCTGGCACAAC ACCTTTCTTCCACGGACTTCTG	91
CADM1	XM_017017461	GCTAAAAGGCAAATCGGAGGTG TTGTGCACCTTCAGCATCAG	74
GAPDH	NM_002046	TCAGCCGCATCTTCTTTTGC ACGACCAAATCCGTTGACTC	90
ACTB	NM_001101	AAAGACCTGTACGCCAACAC CTCAGGAGGAGCAATGATCTTG	132
HPRT	NM_000194	TGGCGTCGTGATTAGTGATG ACATCTCGAGCAAGACGTTC	141

## Results

### The general outcomes of the microarray analysis

From the whole transcriptome, consisting of 2579 different genes, 626 were up-regulated and 831 were down-regulated after 7 days of culture, 829 were up-regulated and 936 were down-regulated after 15 days of culture, while 926 were up-regulated and 1126 were down-regulated after 30 days of culture. Differences were usually only observed between day 1 and the further periods of culture.

**Fig. 1:**
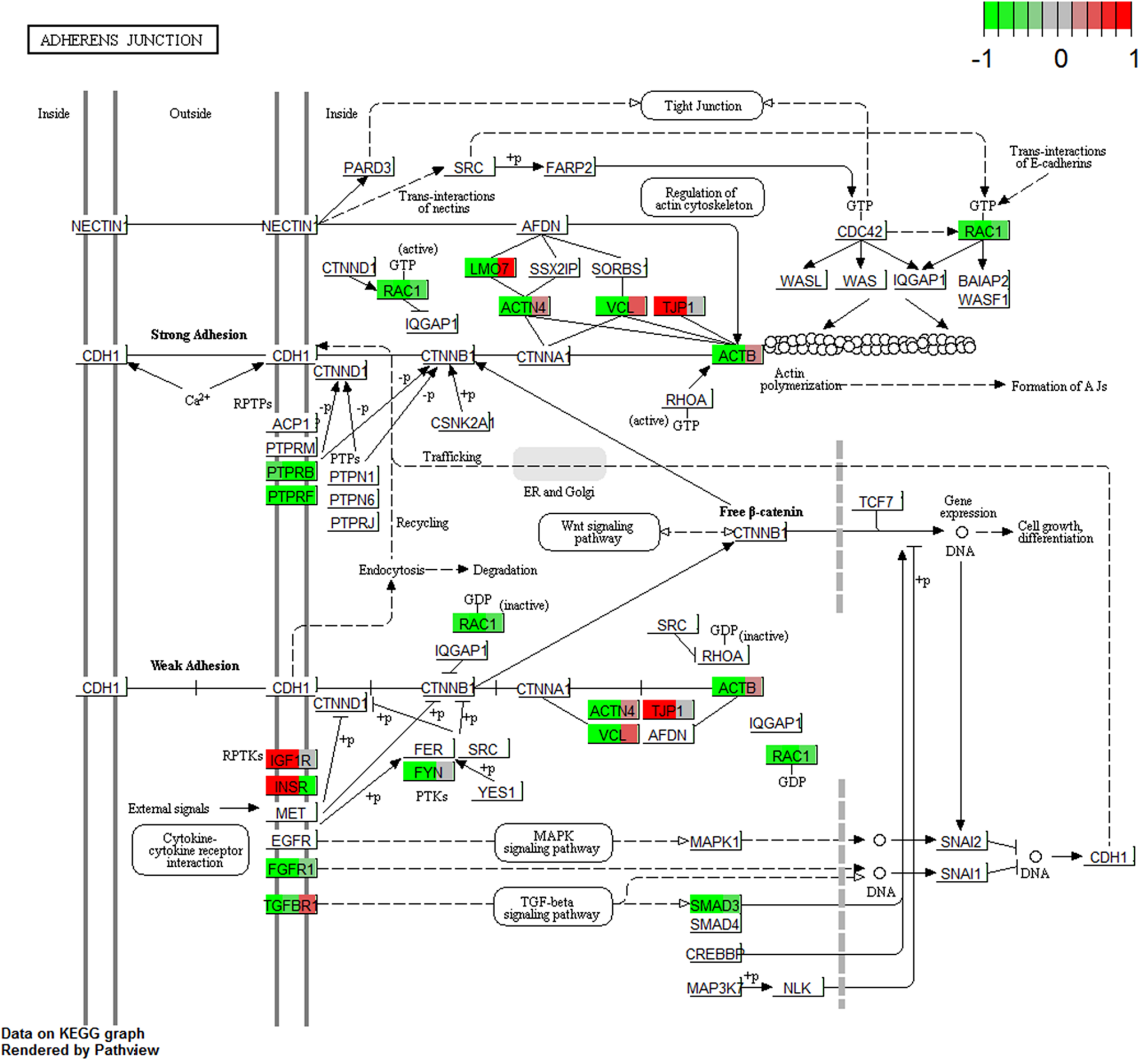
The “adherence junction” KEGG pathway with marked expression levels of differently expressed genes. Arbitrary signal intensity, acquired from microarray analysis, is represented by colours (green, higher; red, lower expression). The boxes with names of genes were separated into three parts containing the representation of gene expression from the 7th, 15th and 30th day of culture

**Fig. 2:**
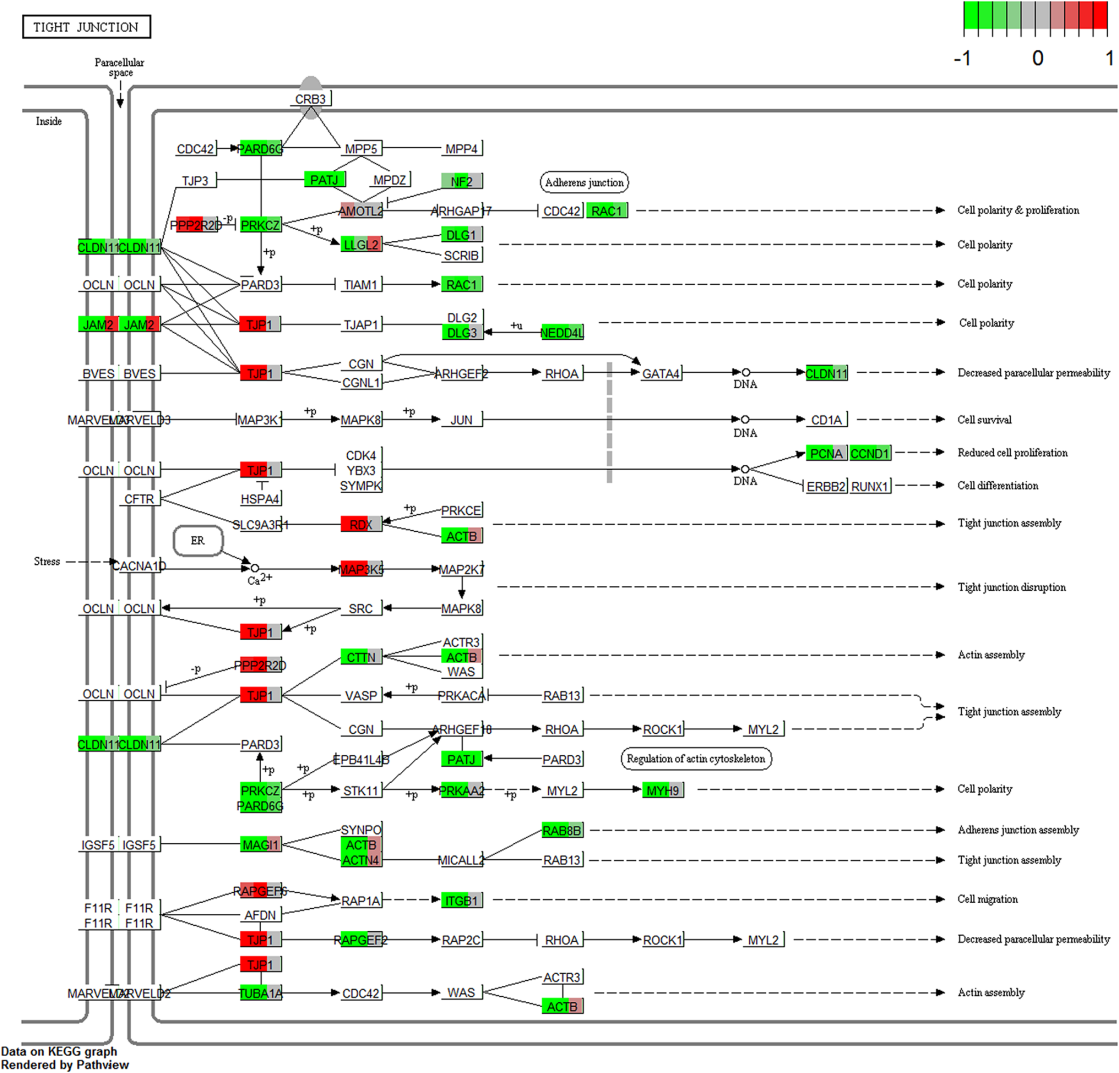
The “tight junction” KEGG pathway with marked expression levels of differently expressed genes. Arbitrary signal intensity, acquired from microarray analysis, is represented by colours (green, higher; red, lower expression). The boxes with names of genes were separated into three parts containing the representation of gene expression from the 7th, 15th and 30th day of culture

The DAVID software analysis showed that the differentially expressed genes belong to 582 Gene Ontology groups and 45 KEGG pathways. In this paper, we focused on genes which belong to the “adherens junction” and “tight junction” pathways from Kyoto Encyclopedia of Genes and Genomes (KEGG), as well as to “cell differentiation” (GO:0030154), “cell proliferation” (GO:0008283) and “cell–cell junction organization” (GO:0045216) gene ontology groups. Up- and down-regulated gene sets were subjected to the DAVID search separately. Only the gene sets where adj. *p* value was lower than 0.05 were selected. Subsequently, the expression levels of genes belonging to “adherence junction” and “tight junction” KEGG pathways were marked on the graphs, using the “path view”—a toolset for pathway based data integration and visualization (Luo and Brouwer 2013) (Figs. 1, 2). The fold changes and adj. *p* values of those genes are shown in Tables 2 and 3.

**Table 2:** Differentially expressed genes belonging to “adherence junction”

Official gene symbol	foldD7_D1	foldD15_D1	foldD30_D1	adj. *p*. value D1_D7	adj. *p*. value D1_D15	adj. *p*. value D1_D30	Entrez Gene
TJP1	0.3867947	0.3770188	0.4099497	0.0130768	0.0106094	0.0124798	7082
INSR	0.3873414	0.8061633	0.5346824	0.0490067	0.6204884	0.124498	3643
IGF1R	0.4564233	0.4255477	0.3166935	0.0747752	0.052006	0.0193157	3480
PTPRB	1.3851168	10.794593	2.9568251	0.7074275	0.0155286	0.1342982	5787
RAC1	1.5261235	2.052283	2.3791522	0.1905251	0.0425477	0.0220353	5879
FGFR1	1.7839941	2.1815486	3.4704025	0.1721854	0.0725808	0.0163142	2260
SMAD3	1.977835	2.6686384	2.3233728	0.0746144	0.0227799	0.0327692	4088
ACTB	1.9832451	1.6575768	2.1164596	0.0178824	0.0395711	0.0106134	60
TGFBR1	2.1889956	1.5854182	1.4680103	0.026412	0.1065456	0.1556766	7046
ACTN4	2.5842058	2.3022849	2.6961029	0.0016345	0.0021662	0.0010491	81
PTPRF	2.7028776	4.7708528	4.2652909	0.0310595	0.0069323	0.0076285	5792
FYN	2.7655141	2.90417	2.6326987	0.0257378	0.0200935	0.0242376	2534
ACTN1	3.6591712	3.1534043	3.6697117	0.0008244	0.0010905	0.0006175	87
VCL	4.8645726	3.3807533	5.783599	0.020792	0.0407899	0.0119742	7414
LMO7	28.286136	14.459734	24.18382	0.0015722	0.002502	0.0012393	4008

Fold changes, adjusted *p* values and Entrez Gene ID of differentially expressed genes belonging to the “adherence junction” KEGG pathway. Symbols and names of the selected genes are also shown

**Table 3:** Differentially expressed genes belonging to “tight junction”

Official gene symbol	Fold change D7/D1	Fold change D15/D1	Fold change D30/D1	Adjusted *p*. value D7/D1	Adjusted *p*. value D7/D15	Adjusted *p*. value D7/D30	Entrez Gene ID
MAP3K5	0.2131741	0.2378015	0.1291998	0.005982176	0.006818764	0.002109835	4217
RDX	0.3062443	0.2730968	0.1285235	0.022738313	0.015307731	0.003275229	5962
AMOTL1	0.3434056	0.3890707	0.4316029	0.00439234	0.00587025	0.007212816	154,810
TJP1	0.3867947	0.3770188	0.4099497	0.013076762	0.010609413	0.012479828	7082
PPP2R2D	0.5926332	0.5781669	0.487322	0.050872315	0.0395242	0.015491178	55,844
RAPGEF6	0.6687086	0.6331767	0.4846757	0.126146642	0.080651203	0.018910566	51,735
NF2	1.2577307	1.2366857	2.0932489	0.376372755	0.39067947	0.017972215	4771
RAC1	1.5261235	2.052283	2.3791522	0.190525052	0.042547728	0.022035289	5879
DLG1	1.6730427	1.8150529	2.8723941	0.235467987	0.155379921	0.030426109	1739
ITGB1	1.8128698	1.8176084	2.2358846	0.024443274	0.021895925	0.007582053	3688
CTTN	1.8861615	1.90003	2.0347218	0.002853089	0.002578576	0.001595196	2017
PARD6G	1.9298224	2.5969128	2.8963465	0.137910778	0.044816837	0.029599738	84,552
ACTB	1.9832451	1.6575768	2.1164596	0.017882382	0.039571145	0.010613354	60
AMOTL2	2.2510959	1.753789	2.2826105	0.024608663	0.064922906	0.01880137	51,421
PCNA	2.3001665	2.4071855	2.0575267	0.017040231	0.012566848	0.021038161	5111
TUBA1A	2.3402647	2.1361513	2.3468717	0.012904376	0.016074104	0.010088367	7846
LLGL2	2.3772916	1.6361034	1.2549524	0.032817316	0.138611913	0.474636591	3993
PRKCZ	2.4168219	3.2661663	2.7576197	0.068631714	0.026079366	0.037507268	5590
DLG3	2.4369791	2.551924	2.641258	0.021918948	0.016637516	0.013295248	1741
INADL	2.5617955	4.536614	3.9061643	0.040584489	0.008377266	0.010444005	10,207
ACTN4	2.5842058	2.3022849	2.6961029	0.001634517	0.002166184	0.0010491	81
RAPGEF2	2.699117	2.7100365	2.220224	0.014946734	0.012876664	0.023381943	9693
MAGI1	2.8485172	2.1931007	1.7717224	0.022590036	0.047934298	0.105439274	9223
MYH9	3.0488704	3.3796457	3.3547395	0.008217194	0.005622839	0.004944816	4627
NEDD4L	3.4767112	4.9176721	3.0807669	0.009286098	0.003901591	0.01001863	23,327
ACTN1	3.6591712	3.1534043	3.6697117	0.000824408	0.001090539	0.000617487	87
PRKAG2	4.2205127	2.9365982	6.3430763	0.005788171	0.012549391	0.00219352	51,422
RAB8B	4.7774458	5.8711379	4.6905655	0.001382959	0.000911951	0.000986744	51,762
MYL9	4.8382842	3.9460484	4.9556207	0.005948833	0.008193716	0.004406837	10,398
CCND1	5.1728662	6.88934	14.6717331	0.055264324	0.030668127	0.009914025	595
MYH10	5.3834258	6.30372	6.2832283	0.075953296	0.052311615	0.048234636	4628
PRKAA2	8.2559913	10.563523	8.1611131	0.041708429	0.026938194	0.034475365	5563
JAM2	21.0028875	13.5493573	9.8219339	0.015282557	0.021967062	0.029572679	58,494
CLDN11	50.2474792	59.0106475	68.9834723	0.01480309	0.01157606	0.009130531	5010

Fold changes, adjusted *p* values and Entrez Gene ID of differentially expressed genes belonging to the “tight junction” KEGG pathway. Symbols and names of the selected genes are also shown

**Fig. 3:**
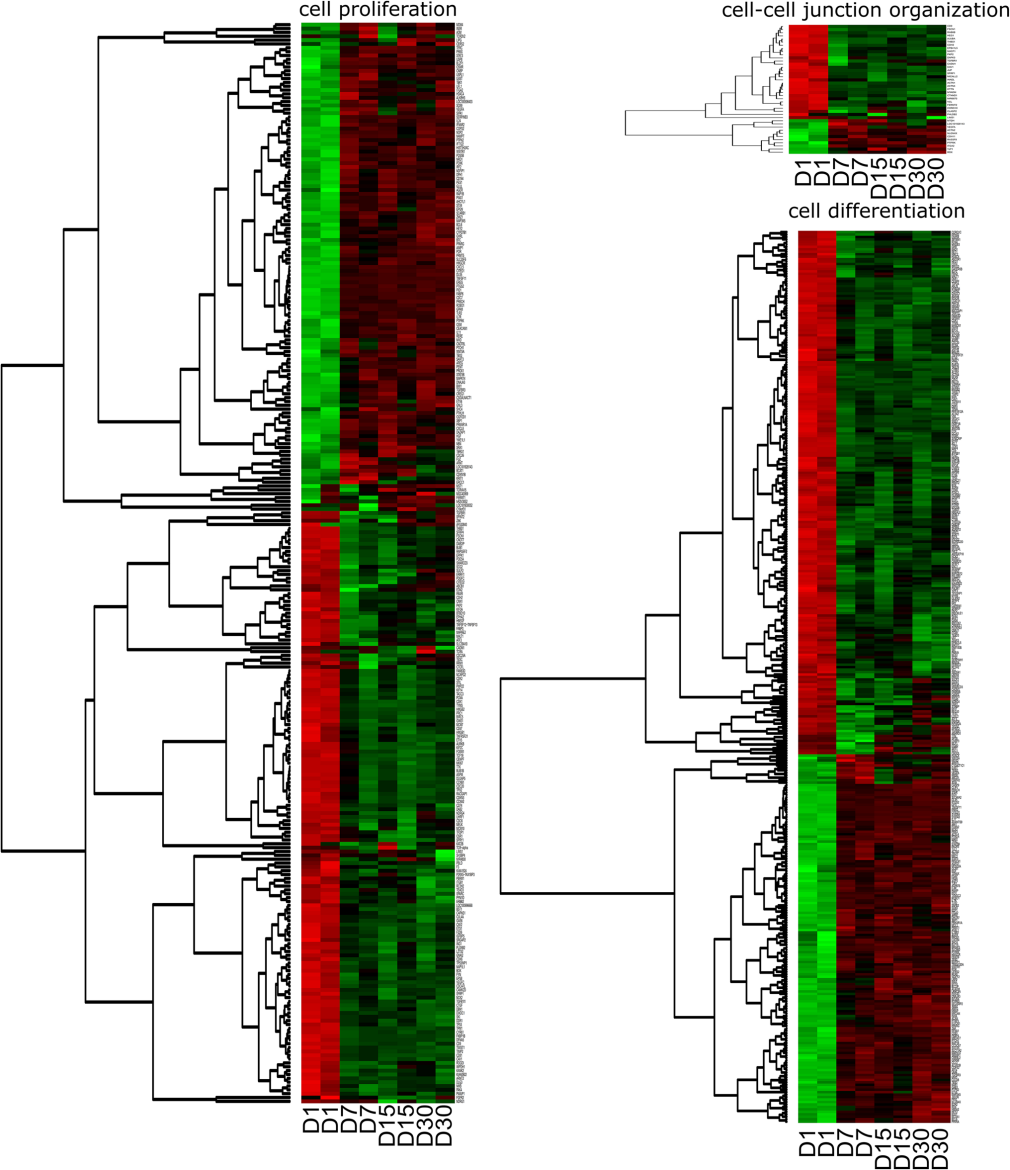
Heatmap representation of differentially expressed genes belonging to the “cell differentiation” (GO:0030154), “cell proliferation” (GO:0008283) and “cell–cell junction organization” (GO:0045216) gene ontology groups. Arbitrary signal intensity, acquired from microarray analysis, is represented by colours (green—higher expression; red—lower expression). log2 signal intensity values were resized to Row *Z* Score scale for all of the genes (from − 2, the lowest expression to + 2, the highest expression)

Selected GO BP terms were subjected to hierarchical clusterization procedure and presented as heatmaps (Fig. 3). Fold changes in expression of all analyzed genes, their Entrez IDs, and adjusted *p* values were shown in Supplementary Table 1.

**Fig. 4:**
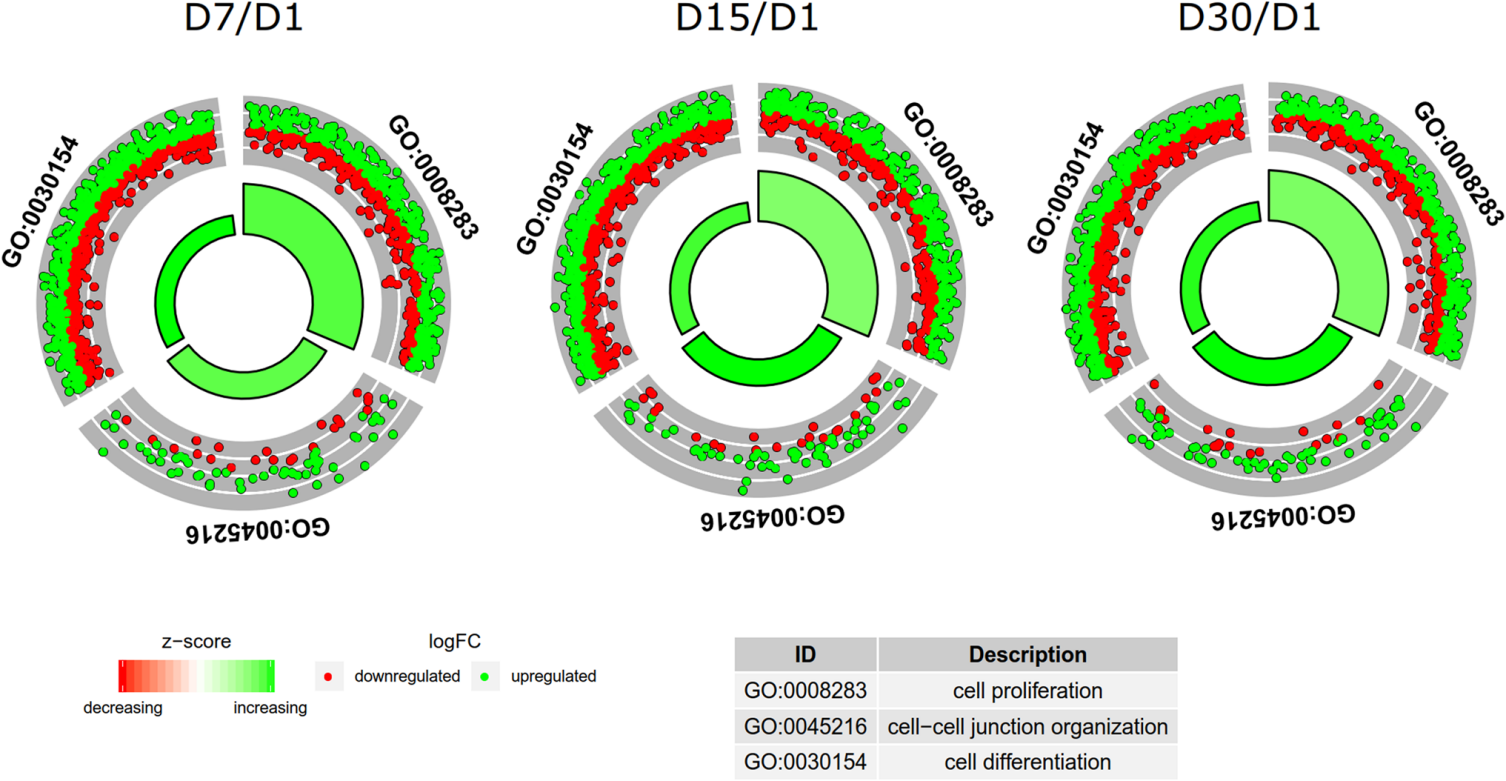
The circular visualization of the results of gene-annotation enrichment analysis. The outer circle shows a scatter plot for each term of the logFC of the assigned genes. The green dots display up-regulation, with the red ones representing down-regulation. The inner circle is a representation of the *z*-scores of respective ontology groups

To further investigate the changes within the chosen GO BP terms, the enrichment levels of each selected GO BP term were measured. The enrichment levels were expressed as z-scores and presented as a circular visualization (Fig. 4).

### Further analysis of microarray

**Fig. 5:**
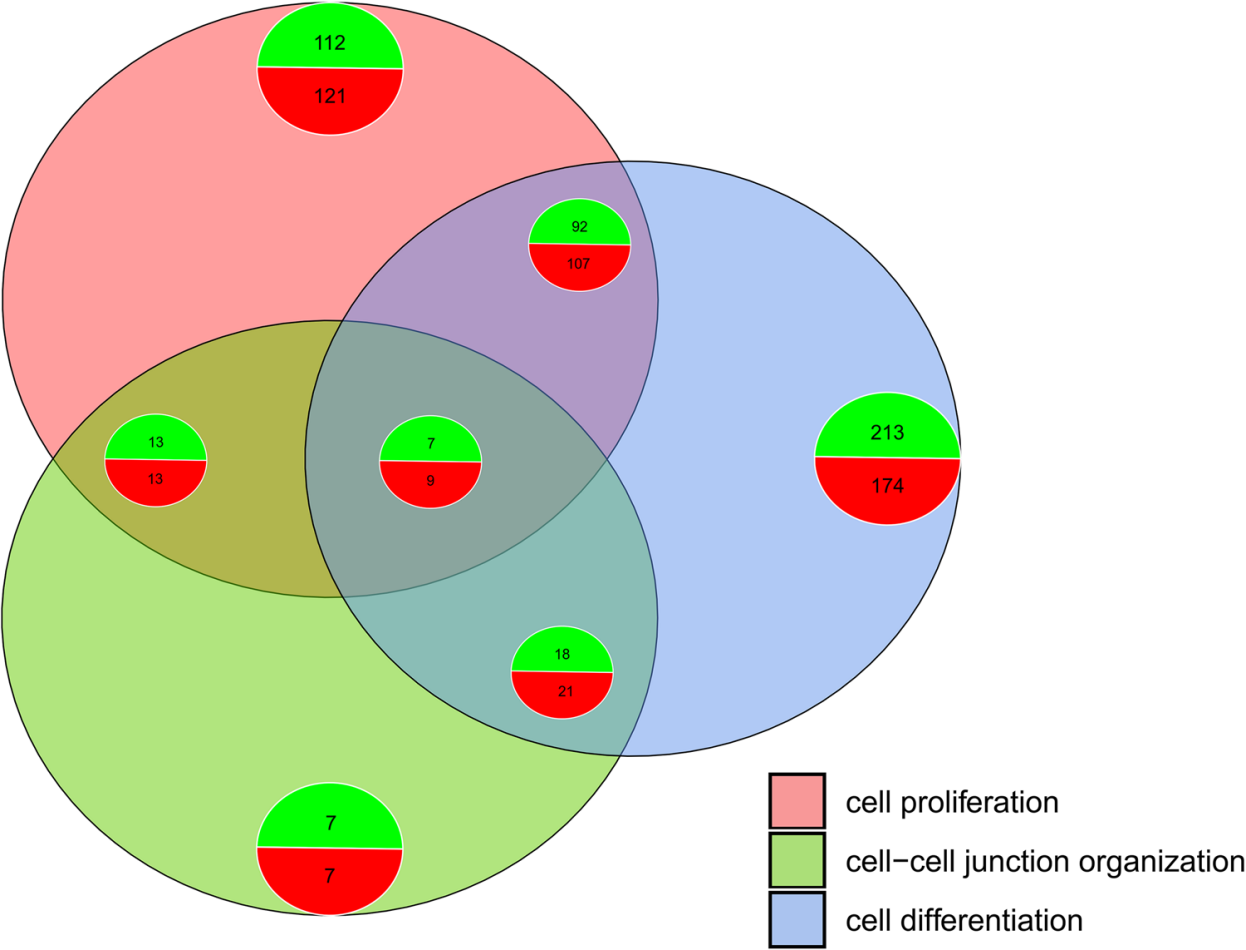
The Venn diagram displaying the number of overlapping genes within “cell differentiation” (GO:0030154), “cell proliferation” (GO:0008283) and “cell–cell junction organization” (GO:0045216) gene ontology terms. The diagram also provides the information about the gene expression patterns (up-regulated—green, down-regulated—red), with the intensity of the colours indicating the levels of expression changes

**Fig. 6:**
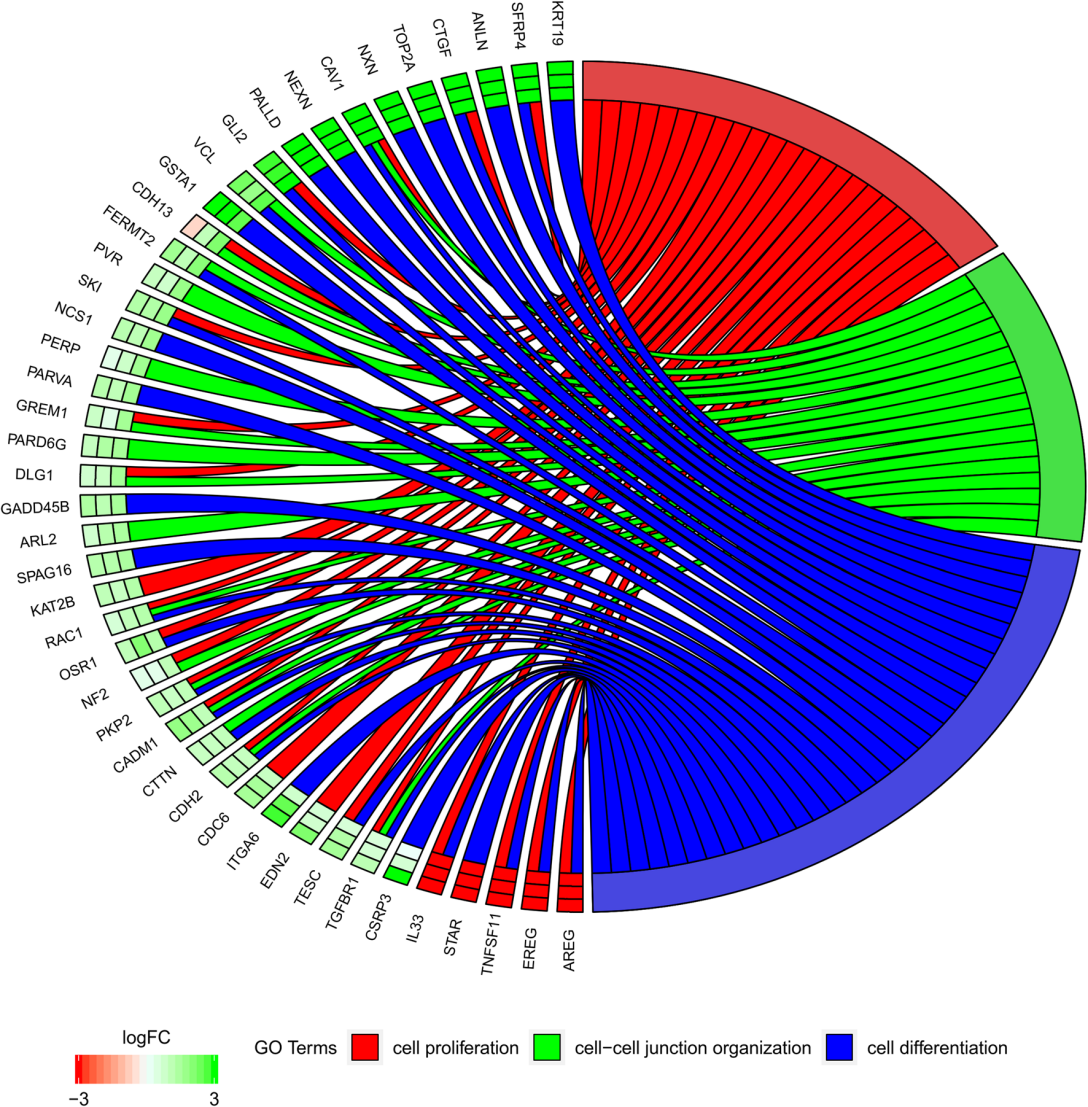
The representation of the relationship between genes belonging to “cell differentiation” (GO:0030154), “cell proliferation” (GO:0008283), and “cell–cell junction organization” (GO:0045216) gene ontology terms. The ribbons show which gene belongs to which category

**Fig. 7:**
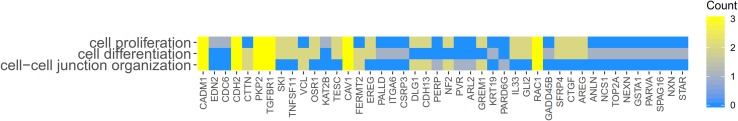
The heatmap of the relationship between the genes belonging to “cell differentiation” (GO:0030154), “cell proliferation” (GO:0008283), and “cell–cell junction organization” (GO:0045216) gene ontology terms. Biological processes are displayed in rows, with genes presented in columns. Each column is divided into smaller rectangles, with the colouring of the tiles depending on the presence or absence of the gene within the GO term

In the Gene Ontology database, genes that formed one particular GO group can also belong to other different GO term categories. For this reason, the gene intersections between the selected GO BP terms were explored. The exact amount of up-regulated and down-regulated genes that are shared within GO BP terms were presented as a Venn diagram (Fig. 5). Subsequently, to focus on selected genes, the mutual relation between GO BP terms was investigated. The results are presented as a circle plot (Fig. 6) as well as heatmap (Fig. 7).

**Fig. 8:**
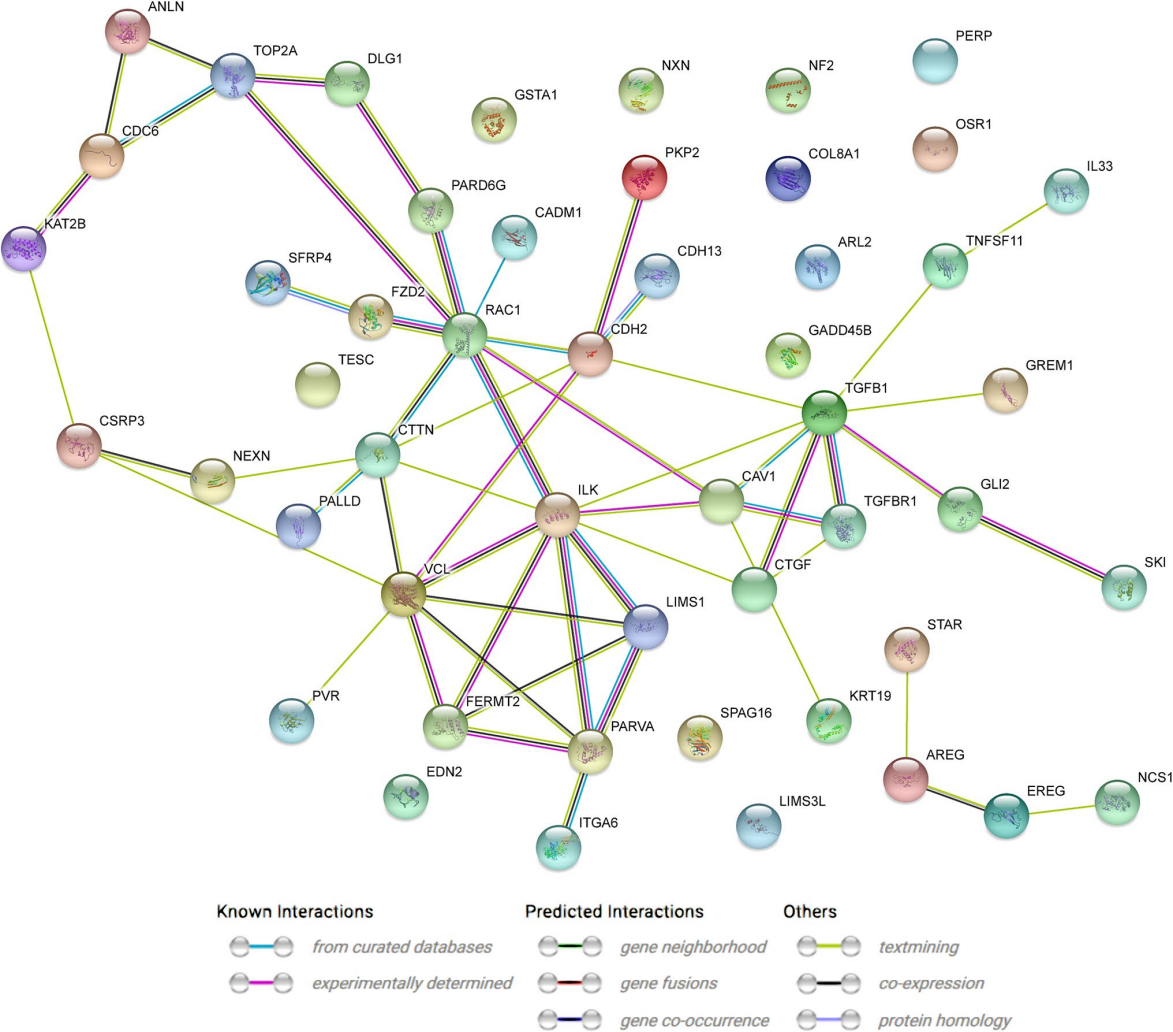
STRING-generated interaction network between differentially expressed genes chosen for further analysis. The intensity of the edges reflects the strength of the interaction scores

Of all the genes representing the above ontological groups, the following were chosen: *VCL, FERMT2, LIMS1, ILK, PINCH1, TGFB, CDH2, PARVA, FZD2, NCS1, SKI, GLI2, KAT2B, COL8A1, CSRP3, GADD45B, SPAG16, OSR1, ITGA6, EDN2, TESC, TGFBR1, CDC6, PKP2, CADM1, CAV1, PALLD, NXN, NEXN, CTGF, TOP2A, GSTA1, KRT19, ANLN, SFRP4, CDH13, PERP, NF2, RAC1, ARL2, DLG1, GREM1, CTTN, PVR, PARD6G, EREG, TNFSF11, STAR, AREG, IL3*. The STRING figure presents the analysis of interactions between the selected genes (considered the most important in the processes related to GC differentiation and proliferation, used for validation of results, and exhibiting the highest and lowest expression) (Fig. 8).

### Real-time qPCR validation

**Fig. 9:**
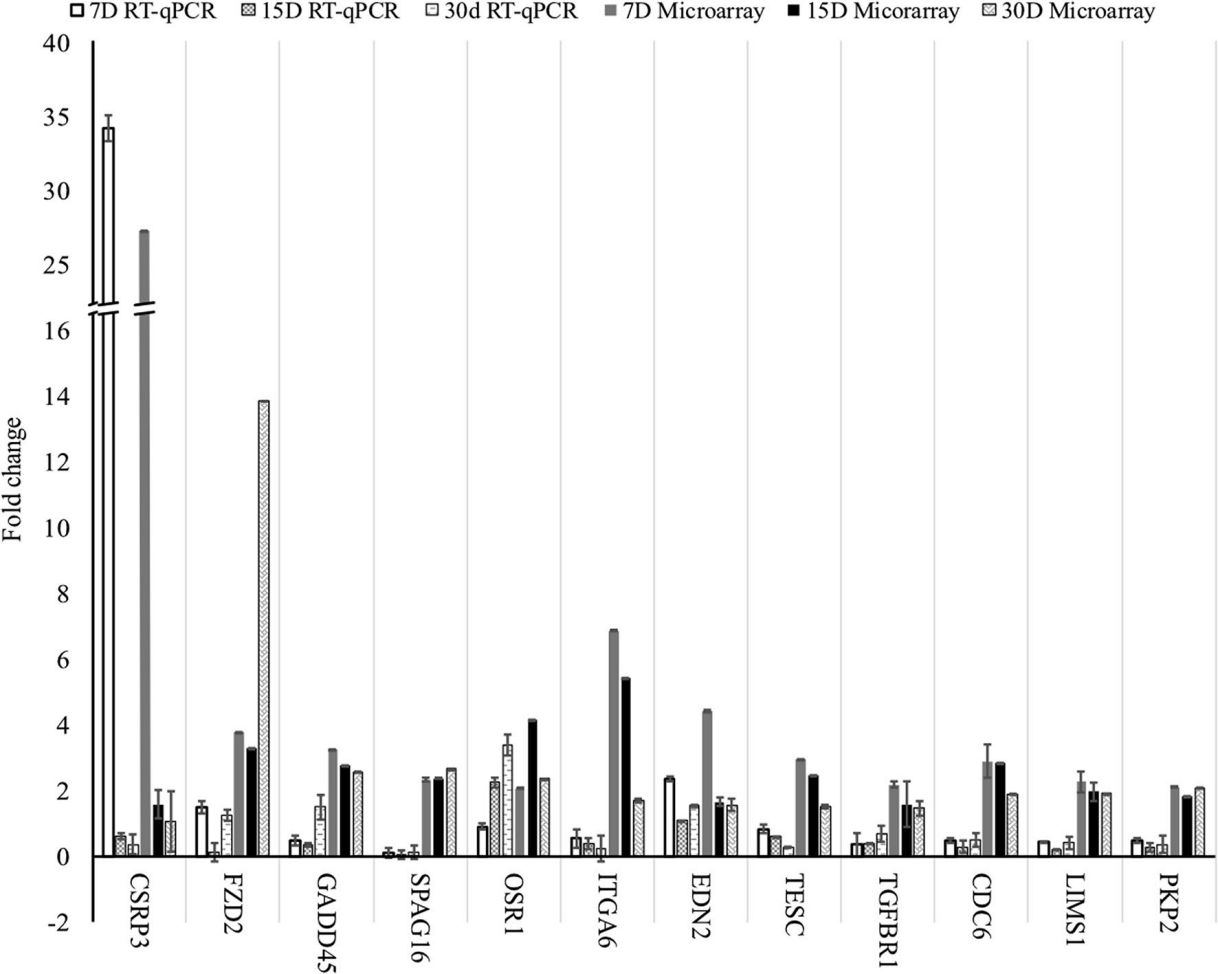
Results of the RT-qPCR validation presented in the form of a bar chart, with comparisons to the results obtained through the microarray analysis. All the values presented are the relative changes in gene expression, compared to Day 1 of primary culture. *D* day of culture

RT-qPCR reactions were used to validate the microarray results. Examples of genes exhibiting each expression pattern that showed the biggest (*CSRP3, FZD2, GADD45B, EDN2, LIMS1*), smallest (*SPAG16, TESC, TGFBR1, PKP2*), and the most intermediate (*OSR1, ITGA6, CDC6, CADM1*) changes in expression, were chosen. Overall, the changes in expression of the analyzed gene transcripts were confirmed in every example. However, while the qualitative validation was a success, the quantitative results were not always corresponding between RT-qPCR and microarrays. Obtained values were presented in a form of a bar graph (Fig. 9).

### Changes in morphology of granulosa cells

**Fig. 10:**
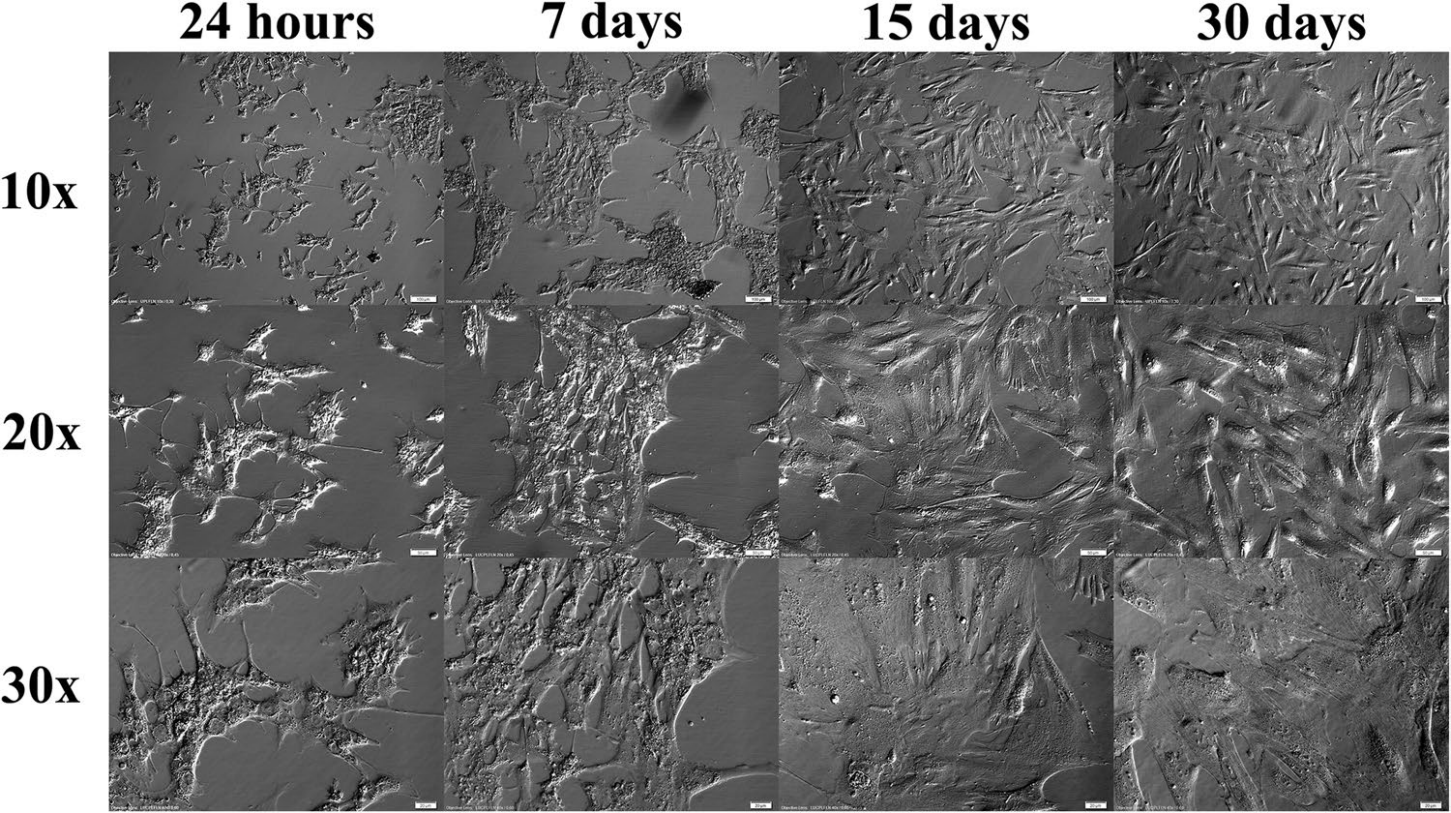
Changes in morphology of granulosa cells during the long-term culture

A substantial change in GCs’ morphology was observed during the long-term primary in vitro culture. Multiple cultures were performed at the same time, with the majority of them presenting comparable morphology changes which, in our opinion, deemed the GCs viable for our research. The alterations in morphology were observable, as the cells changed their morphology from epithelial-like to fibroblast-like during the time of the culture (Fig. 10).

### Immunofluorescence analysis of LHR and FSHR expression

Immunofluorescence analysis of LHR and FSHR localization and distribution in cultured GCs showed that expression of receptors decreased during long-term in vitro culture (Suppl. Figure 1). It was clearly observed at 1 day and then gradually diminished, being slightly visible on the 7th day and totally negative on day 30. This proves that GCs are initially present in the culture but change their character and gain new properties during long-term cultivation.

## Discussion

GCs play an important role in the formation of the ovarian follicle. It is known that, in addition to the hormonal secretory function, they also play an important role in communication with oocytes. It is possible thanks to proteins called connexins, forming gap-junctions connections (GJCs), enabling the transfer of nutrients and stimulating agents between GCs and the oocyte (Kempisty et al. 2014a). This has been demonstrated by many examples on various animal models, including pig (Anderson et al. 2009; Kranc et al. 2017b; Truman et al. 2017).

In recent years, an interest has grown in exploring the differentiation potential of various tissue types, especially that of present populations of cells with stem-like features. These processes involve the differentiation of less specialized cells into more counterparts, with their subsequent, progressive lineage commitment (Avior et al. 2016). It was suggested that GCs exhibit stem-like properties during long-term IVC. In a pioneering research, Kossowska-Tomaszczuk et al. suggested that GCs may differentiate into osteoblasts, chondrocytes, and neuroblasts under the influence of appropriate differentiating factors (Kossowska-Tomaszczuk et al. 2009). Other studies have shown that GCs can also differentiate toward muscle cells (Brevini et al. 2014).

The aim of this article was to present new, poorly understood properties of GCs. Our research has revealed gene ontology groups characterizing proliferative and differentiative potential of the granulosa. However, no supplementation with differentiating factors was used. Because of that, we cannot indicate the specific direction of differentiation. Hence, we have only selected and highlighted the genes that were possibly related to the GC differentiation process, being aware of their described potential to differentiate towards other cell types (Kossowska-Tomaszczuk et al. 2010; Brevini et al. 2014) (main functions of selected genes have been summarized in Supplementary Table 2).

The presented research combines the analysis of changes that occur during long-term in vitro culture of human GCs. These include morphology, expression of GC characteristic receptors (LHR and FSHR), as well as expression of genes characteristic for cell proliferation and differentiation.

Our first observation clearly demonstrated significant alterations in the morphology of cultured GCs, staying in accordance with the results of several previous studies. It was well-documented that, at the beginning of IVC, the GCs usually present a epithelial-like structure. Then, after several weeks of culture, accompanied by reaching full confluency, the cells adopt fibroblast-like morphology (Quinn et al. 2006; Oki et al. 2012).

Another change was related to the decreasing expression of LH and FSH receptors on the surfaces of analyzed cells, during the time of culture. The major difference was seen after the seventh day of culture when LHR and FSHR expression was almost completely lost and confirmed on day 30 when the result was totally negative. A similar observation was made by the research group of Kossowka-Tomaszczuk. However, their GCs lost FSHR expression after 17 days of culture (Kossowska-Tomaszczuk et al. 2010). Interestingly, our GCs kept proliferating until the 30th day of IVC. This fact is surprising, keeping in mind that under physiological conditions, after about 10 days post-ovulation, GCs turn into the corpus luteum and then into an inactive corpus albicans, ending their proliferation (Niswender et al. 2000; Stocco et al. 2007). Taking these observations together, we can assume that GCs acquire new properties under long-term in vitro conditions, possibly including the potential for differentiation towards new cell types, which emphasizes their possible role in the future of tissue engineering.

Apart from morphological alterations, we have found that GCs presented significant changes in expression of genes, belonging to the “cell proliferation”, “cell differentiation” and “cell–cell junction organization” ontological groups, during the time of the culture. This large cohort of genes is responsible for processes in which less specialized cells, such as those with regenerative potential, acquire specialized features (functional or structural) specific to the fully developed tissue (Dzafic et al. 2013). Although we could distinguish several patterns of expression changes, we focused on genes characterized by significant up-regulation after day 1 of culture.

First of them, which attracted our attention, was *FZD2* (*FRIZZLED CLASS RECEPTOR 2*). Expression of *FZD2* is detectable in developing ovaries (Zhao et al. 1995). The function of this gene, in physiological condition, is not fully understood, but most likely it is involved in the transmembrane signal transmission. Being a member of the WNT/β-catenin signalling pathway, it could play a significant role in developmental processes involving rapid cell proliferation and differentiation. Studies on human GCs suggest that WNT2 can regulate β-catenin pathways and influence folliculogenesis via FZD receptors (Wang et al. 2009). Additionally, Wang et al. found the highest FZD2 mRNA and protein level in murine oocytes and granulosa cells during the proestrus phase, observing a decrease compared to oestrus to diestrus (Wang et al. 2010). Since all of the collected COCs in our study were in the proestrus stage, increasing expression of this gene stays in accordance with this data. Moreover, its ability to regulate cell differentiation has been confirmed by our results of increased gene expression during long-term culture of GCs, underlining possible new functions of *FZD2*.

The differentially expressed genes analyzed in our study also included *VCL* (*VINCULIN*), a gene responsible (in physiological conditions) for the synthesis of a cytoskeletal protein associated with F-actin in the cell membrane (Weller et al. 1990; Silva et al. 2005; Israeli-Rosenberg et al. 2014). It has also been shown that VCL is a significant contributor to the GC cytoskeleton formation and is associated with the modulation of cytoskeletal proteins, potentially playing a key role in the differentiation of GCs into their particular types during folliculogenesis. So far, studies have shown that rat GCs under the influence of insulin, FSH and chorionic gonadotropin (HCG), increased expression of *VCL*, revealing that it leads to a higher level of steroidogenesis, and lower organization of microfilaments, vinculin and actin (Kranen et al. 1993). Moreover, high expression of *VCL* in osteoprogenitor cells plays an important role during the proliferation and differentiation of osteoblasts. *VCL* is critical for creating the actin skeleton and regulating cell adhesion during osteodifferentiation (Hong et al. 2010). It is suggested that *VCL* may be responsible for the final phenotype of GCs (Ben-Ze’ev and Amsterdam 1987). Our observations revealed that *VCL* may also affect the changes in the cytoskeleton of GCs during their differentiation into other cell types. This assumption was made based on our observation of increased expression of *VCL* in human GCs during long-term IVC without the addition of hormones (FSH, HCG, and differentiation stimulating factors).

Another gene that is also related to the developmental processes, is the *SKI* gene (*ONCOGENE SK*). Multiple studies of Kim et al., performed on rats, presented the evidence that *SKI* expression is observed in atretic follicles, but not in preovulatory follicles. This suggests that the gene can play a role in apoptosis of the GCs. On the other hand, granulosa cells undergo substantial differentiation towards luteal cells, which points out the role of this gene in their transformation (Kim et al. 2012). This note, together with our observation of increased expression, suggests that *SKI* can also play a role in differentiation of GCs towards other cell types. Previous data describes the *SKI* expression in the embryo during the development of blood vessels—especially the aorta, and suggests that this gene is involved in the regulation of TGF-β signalling in arterial media during embryonic development (Doyle et al. 2012). This information justifies the recent finding of Basini et al., who presented the evidence that GCs cultured in endothelial culture medium (EBM-2) gained functional and phenotypic characteristics of endothelial cells (Basini et al. 2016).

Cell proliferation depends partly on proper cell communication. One of the genes associated with these processes is *CDH2* (*CADHERIN 2*). It belongs to the family of genes encoding calcium ion-dependent adhesion proteins. Studies on hamster ovarian follicles showed that, while expression of *CDH1* is restricted only to oocytes during neonatal ovary development, *CDH2* is present in the GCs of growing follicles (Wang and Roy 2010). Additionally, cadherin 2, together with vimentin, is a marker of GCs mesenchymal origin. Our observation of increased expression of *CDH2* during long-term IVC proves that GCs maintain their mesenchymal character, without shifting towards epithelial phenotype (Yenuganti and Vanselow 2017). In spite of not knowing the exact direction of GCs differentiation, it is established that *CDH2* also participates in the developmental process of the nervous system and formation of cartilage and bone (Chung et al. 2014). Up-regulated expression of the above gene may suggest the potential of GC differentiation towards osteoblasts. Moreover, high expression of the *K(lysine) Acetyltransferase 2B* (*KAT2B*) gene may also indicate the differentiational potential of the GCs, since it activates the *RUNX2* transcription factor, which promotes mesenchymal stem cell differentiation towards osteoblasts (Dzafic et al. 2014). The above gene is responsible for the production of nuclear proteins that bind many factors, characterizing cell growth and differentiation (Yang et al. 1996).

Another gene associated with the cellular communication is *NCS1* (*FREQUENIN*). Signal transmission can take place in multiple ways, including those that involve neurotransmitters (Hancock 2017). The demonstrated study suggests that expression of the *NCS1* gene (*FREQUENIN*) is increasing during long-term IVC. This gene encodes a protein that acts as a calcium ion sensor, modulating synaptic and secretory activity (De Castro et al. 1995). Mc Ferran et al. suggests that *NCS1* is primarily expressed in neuroendocrine cells and may be responsible for the regulation of neurosecretion (Burgoyne and Weiss 2001). Although we could not find direct evidence, up-regulated expression of this gene may indicate its role in GCs differentiation towards neuronal cells. This would stay in accordance with results of Kossowska-Tomaszczuk, who proved that GCs can acquire features of neuroblasts under influence of the differentiating factors.

Cellular viability and proliferation are associated with transforming growth factor beta (TGF-β) (Juengel et al. 2004). TGF-β exhibits a variety of biological activities and is a potential regulator of cell proliferation and differentiation (Tripurani et al. 2013; Ribeiro et al. 2017). Referring the literature data to the obtained results, it may be noted that these genes may also influence proliferation and cell viability during in vitro culture. Under physiological conditions, GCs form a corpus luteum, which transforms into corpus albicans after around 10 days. Under in vitro culture conditions, these cells remained viable up to day 30 of culture. Expression of the mentioned genes was also maintained. Studies show that different types of TGF-β receptors (TGFBR) exist on the cell surface. TGFBR are either transmembrane receptors or cytoplasmic tyrosine kinases (Kingsley 1994). We found increased expression of *TGFB* and *TGFBR1* (*TYPE I TGF-β RECEPTOR*) during the time of IVC. This receptor is involved in the induction of some genes involved in the cell–matrix interaction (Ebner et al. 1993). In the pig model, Paradis et al. clearly indicated that TGF-β and its receptors play an important role during ovulation. These factors support the normal/correct action of BMP15 and BMPR1B (Paradis et al. 2009). Moreover, since it is a very strong oncogene, *TGFBR1* promoted granulosa cell tumour development in mice (Gao et al. 2017). Overexpression of *TGFBR1* also contributes to morphological, hormonal and molecular changes in GCs (Gao et al. 2016). Additionally, *TGFBR1* is a gene characterizing human placental mesenchymal stem cells (Abumaree et al. 2013). The presented studies suggest that the above genes play a key role not only in the proliferation of GCs under physiological conditions but also during cell proliferation and differentiation during long-term in vitro culture.

Apart from genes which can be directly linked to the physiology of GCs, we analyzed those, whose role in folliculogenesis and/or cells differentiation has not yet been described. Based on available data, we tried to interpret our results and find the potential role of described genes in the process of granulosa cell differentiation.

Among them was *PARVA* (*PARVIN ALPHA*), increased expression of which has so far been detected in the heart, skeletal muscles, kidneys and liver (Korenbaum et al. 2001). Parvin belongs to the family of proteins involved in the integration of proteins which is involved in the intracellular pathways responsible for the dynamics of the actin cytoskeleton and cell viability. Parvin forms a complex with ILK (Integrin-linked Kinase) and LIMS1 (LIM And Senescent Cell Antigen-Like Domains 1) proteins, playing a key role in cell’s protection against the apoptotic process, promoting cell survival and viability (Fukuda et al. 2003). Interestingly, we have also noted the up-regulated expression of *LIMS1*. Due to the lack of data describing the role of both genes in the physiology of GCs, we can only assume that this complex may also promote prolonged proliferation and viability of GCs in long-term IVC, as well as serve as an intermediate in GC alterations.

According to recently presented data, GCs can differentiate into few cell types, including chondrocytes (Dzafic et al. 2013; Kossowska-Tomaszczuk and De Geyter 2013; Hummitzsch et al. 2015). Among our analyzed genes, the candidate that could trigger the transition towards chondrocyte lineage is *FERMT2*, also called *Kindlin 2*. Our hypothesis was made based on the fact that *FERMT2* is responsible for regulating the process of chondrocyte differentiation and chondrogenesis. Lack of this gene inhibits TGFB1-induced Smad2 phosphorylation and chondrocyte differentiation (Wu et al. 2015). Consequently, observations of Wu et al. showed that mice lacking *FERMT2* in mesenchymal progenitor cells exhibited high neonatal mortality, chondrodysplasia, and loss of cranial vaulting. Additionally, *FERMT2* is regarded as an integrator and activator, participating in the induction of epithelial–mesenchymal transition (EMT) during embryogenesis and ovulation (Ma et al. 2008). It has been suggested that during ovulation, GCs change their epithelial character and undergo EMT (Yang et al. 2014). The last theory stays in accordance with the outcome of our study, where increased expression of *FERMT2* correlated with the change in GC morphology from epithelial-like to fibroblast-like. In addition, this gene may be responsible for the process of GC differentiation into particular cell types characteristic for mature Graafian follicle (Kossowska-Tomaszczuk and De Geyter 2013).

Some of the differentially expressed genes belonging to the studied ontology groups also confirmed the possibility of GC differentiation into osteoblasts. We can suppose that the transcription factor *GLI2* (*GLI-KRUPPEL FAMILY MEMBER 2*) can be one of them, since it is responsible for the correct development of the spine (Bertolacini et al. 2012). Mutations in the *GLI2* gene cause defects in the development of the skeleton. In addition, *GLI2* has been shown to play a crucial role in regulating BMP-2 protein expression during mesenchymal stem cell differentiation toward osteoblast (Zhao et al. 2006). Similarly, a significant change in expression of *COL5A1* (*COLLAGEN TYPE V ALPHA 1*) has attracted our attention. This gene is thought to be responsible for the proper synthesis of collagen present in the skin, tendons, and bones. Mutations in *COL1A1* were shown to be associated with osteoporosis and other bones disorders (Viguet-Carrin et al. 2006). Although we could not find direct evidence for the involvement of the above genes in the process of osteodifferentiation, we consider this observation as valuable, indicating the possible direction of GC differentiation.

The presented analysis suggests that many genes specific for cell differentiation and proliferation processes show high changes in expression during the long-term primary in vitro culture of the GCs. GCs express both—genes specific to differentiation processes and genes that are usually proprietary to other cell types. It can be assumed that GCs lose their primary function in long-term primary culture and gain new features and qualities. Thus, the above studies suggest the ability of GCs to differentiate towards a range of distinct types of cells and tissues. It is therefore envisaged that a stable culture of muscle cells and/or osteoblasts can be obtained under the influence of specific differentiation factors. The studies presented are intended to show the usefulness of GCs, which are usually only treated as the remnant material of the IVF fertilization. Due to the observed changes in the GCs transcriptome, it may be possible to use these cells in the broadly understood tissue engineering.

## Electronic supplementary material

Below is the link to the electronic supplementary material.

Identification and distribution of FSHR and LHR in human granulosa cells during long-term in vitro culture. Representative images of immunofluorescent reactions performed on the 1^st^, 7^th^ and 30^th^ day of said culture (TIFF 1924 KB)

Fold changes, adjusted *p* values, and Entrez Gene IDs of all the differentially expressed genes detected in this study (XLSX 85 KB)

Supplementary material 3 (DOCX 14 KB)
