# Supplementary figures and images for: Genes responsible for proliferation, differentiation, and junction adhesion are significantly up-regulated in human ovarian granulosa cells during a long-term primary in vitro culture
Source: Histochem Cell Biol. 2018 Oct 31;151(2):125–43. doi: 10.1007/s00418-018-1750-1 (PMC6394675; doi:10.1007/s00418-018-1750-1)

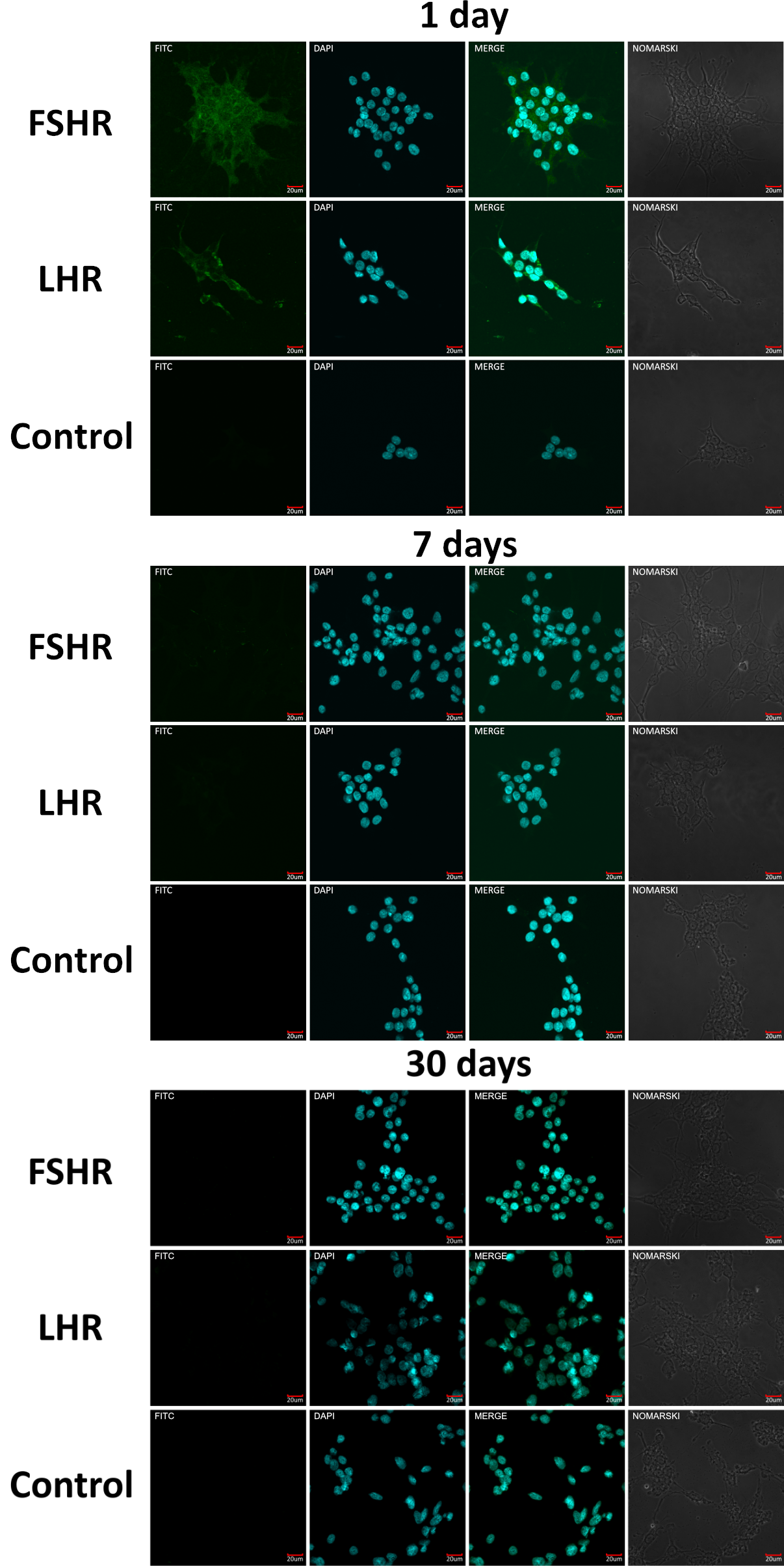

Supplement: Supplementary file 1 — Identification and distribution of FSHR and LHR in human granulosa cells during long-term in vitro culture. Representative images of immunofluorescent reactions performed on the 1st, 7th and 30th day of said culture (TIFF 1924 KB) [file 418_2018_1750_MOESM1_ESM.tiff]
