# Supplementary material for: Genes responsible for proliferation, differentiation, and junction adhesion are significantly up-regulated in human ovarian granulosa cells during a long-term primary in vitro culture
Source: Histochem Cell Biol. 2018 Oct 31;151(2):125–43. doi: 10.1007/s00418-018-1750-1 (PMC6394675; doi:10.1007/s00418-018-1750-1)
Supplement: Supplementary file 3 — Supplementary material 3 (DOCX 14 KB) [file 418_2018_1750_MOESM3_ESM.docx]

Suppl. Tab. 2. Main functions of the genes selected for description in the discussion, together with their fold-changes.

| **Name of gene** | **Symbol** | **Fold change**  **(Day 30/ Day 1)** | **Main selected function** |
| --- | --- | --- | --- |
| *Collagen, type V, Alpha-1* | *COL5A1* | 18,52 | Characterized in human skin, placenta cells |
| *Gli-Kruppel Family Member 2* | *GLI2* | 14,11 | Transcription factor of spine development |
| *Frizzled class receptor 2* | *FZD2* | 13,84 | Promotion of epithelial-mesenchymal transition;  present in developing ovaries, lungs, kidneys |
| *Vinculin* | *VCL* | 5,78 | Cytoskeletal formation |
| *Kindlin 2* | *FERMT2* | 3,97 | Integrator, activator of epithelial-mesenchymal transition |
| *Oncogene SK* | *SKI* | 3,34 | Expression during blood vessels development |
| *Frequenin* | *NCS1* | 3,11 | Regulation of neurosecretion |
| *Parvin Alpha* | *PARVA* | 3,01 | Increase during heart, muscle, liver and kidneys development |
| *LIM And Senescent Cell Antigen-Like Domains 1, PINCH1)* | *LIMS1/PINCH1* | 2,66 | Role in cell proliferation and cell viability |
| *Lysine Acetyltransferase 2B* | *KAT2B* | 2,45 | The highest expression during skeletal muscle and heart development |
| *Transforming growth factor type 1* | *TGFBR1* | 1,48 | Role in ovulation, in cell matrix interaction |
| *Cadherin2* | *CDH2* | 1,92 | Participates in the developmental process, formation cartilage and bone, development of nervous system |
